# Diastase in Therapeutics*Read before the New York Medico-Surgical Society, January 4, 1897.

**Published:** 1897-03

**Authors:** C. C. Fite

**Affiliations:** New York


### DIASTASE IN THERAPEUTICS.*

By C. C. FITE, M.D ,

NEW YORK.

The sprouting of a seed was considered, until quite recent times, a great mystery; not until diastase was discovered could we properly understand why a seed after being placed in a moist soil began to develop into a plant after having lain dormant, perhaps, for years. Under proper conditions the life-endowed germ is no longer a sleeping, unknown quantity, but it is an active principle and the process of development begins.

The sprouting of seed is a very interesting subject for scientific study—this awakening of the germ life, by the influence of heat and moisture, and the action of diastase in converting the starch of the seed into maltose, which afterwards becomes fibrous or cellular tissue. We will not go into a discussion here in reference to the secretion by the plant of fluids which, acting through the delicate roots, absorb and utilize the soil elements; of the important work done by the leaves in taking carbon from the atmosphere; and by these and other processes completing the growth of the plant and eventually a reproduction of seed to continue the life of the species indefinitely.

Let us now leave the plant and refer to the important factor in the growth and development of animal life; the ptyalin of the saliva, a product analogous in many respects to diastase. Just as nature places diastase in the grain to produce the changes leading up to a higher growth, so she gives animal ptyalin to convert the starch and, perhaps, other foods into assimilable material for nourishment, for heat, and a reserve supply of fat. I use the words nourishment, heat, and fat advisedly. The starch so converted is nourishment, and it is the basis of our caloric energy. Our supply of fat comes mainly from the starch changed into maltose by the ptyalin, supplemented by the pancreatic secretions, and it is changed into oil later on in the process. This oil is not only utilized for heat but stored in the tissues as a reserve supply.

* Read before the New York Medico-Surgical Society, January 4, 1897.

It is well to bear in mind that few carnivorous animals take on a great degree of fat; grain-eating animals do. Give swine all of the fat and oils they can eat, and they will not gain in weight half as quickly as when fed on grain alone.

The function of ptyalin is to convert starch into dextrin and maltose, this being the preliminary step, and goes on in the normal stomach for from thirty to forty minutes, after the close of an ordinary meal, when the acid peptic digestion stops the diastasic process. The duodenum holding the pancreatic and other secretions takes up the partially changed starch and completes the conversion. We need not follow the process beyond this point.

It has long been observed that we do not get altogether satisfactory results in the treatment of amylaceous indigestion with pancreatic extracts. This is probably due to the fact that the amylopsin of the pancreatic juice and the other various duodenal enzymes are intended more for completing and finishing the changes already begun by ptyalin before the peptic digestion supervenes, and are not adapted for this preliminary conversion in the stomach. In other words, they are not suitable for beginning the conversion into the food mass as found in the stomach—acting we might say on the mass later when it is an acid body, during the time it is being changed from an acid to an alkaline reaction, instead of on the alkaline or neutral mass when it is being changed into an acid mass. We see, therefore, that white ptyalin and amylopsin are identical in their action on starches. Nature intends them to act under different conditions and at different periods of the digestive process. Therefore, we should not give pancreatic extracts with the expectation that they would render desirable service in the stomach, but we should rely upon either increasing the supply of ptyalin by slowly masticating the food, or by giving a ferment having similar properties to ptyalin; this we have in diastase.

From the discovery of diastase in malt, until quite recently, many attempts have been made to produce it in an isolated form economically enough to be used freely in medicine, without having to give it in the form of the semi-solid malt extracts which have been the only reliable form of it readily obtainable by the profession, as the liquid malt extracts do not contain an appreciable amount of diastase.* The disadvantage in its use in malt extracts is that we have to give a large bulk of the extract to get a very small quantity of diastase ; then too, the extract contains fermentable sugars and extractive matters which may not only be of no value, but may give rise to fermentation in the stomach or intestinal tract.

So far the only chemist, who has succeeded in producing an isolated diastase economically and on a large scale, is Jokichi Takamine, the Japanese chemist. (See the *London Lancet*, May 25, 1896.) This investigator received his scientific education at the Glasgow University. He devoted some years to the study of malting and the production of diastase and other ferments, and upon his return to Tokio was fortunate enough to find that Eurotium oryzae was what he desired. The process he finally perfected is in brief as follows: The seed of the Eurotium is sown on moistened and sterilized wheat bran. The growth is rapid, and after the plant has reached maturity, he calls the bran with the growth on it "Taka-Koji." Upon examining this growth, under the microscope, it is found that the roots of the Eurotium which have penetrated the bran, are covered with crystals of pure diastase. These diastase crystals have the property of converting the starch of the bran for the nourishment of the plant. Takamine named the diastase so produced Taka-diastase. In preparing this diastase for use in medicine it is necessary to get rid of the spore. This is done by percolating the Taka-Koji with water, and adding to this solution of diastase, alcohol, when the diastase is precipitated and the activity of the spores destroyed. It is then a simple matter to further purify the diastase and preserve it in a dry powdered form indefinitely.

This diastase, owing, I presume, to it being an isolated substance, acts much quicker than the diastase found in malt extracts. It will convert 100 times its weight of starch in ten minutes under proper conditions; if the process is continued for three hours, 1,500 times its weight. It is, therefore, evident that its amylolytic-converting power is quite marked, and the theoretical position that it would

*See the *Boston Medical and Surgical Journal* for December 31st, 1896, page 669.

prove of value in the treatment of amylaceous indigestion has been verified by careful observers many times over.

Professor Leo, of Bonn, claims that this diastase exerts its action in a higher degree of acidity than was first claimed by those of us who had experimented with it, and that it is, therefore, superior to ptyalin in its starch-converting power. He has employed it with benefit in cases of deficient salivary secretion, as also in hyper-acidity of the stomach.

Dr. W. S. Christopher, of Chicago (*Therapeutic Gazette,* March, 1896), holds that flatulence is due to micro-organisms which attack unchanged starch and give rise to fermentation, and that it is therefore important in these cases to predigest the starch in the stomach, and the more complete this process, the less food there is for the micro-organisms to act upon in the duodenum. He finds that the administration of diastase in these cases gives satisfactory results.

Dr. R. W. Wilcox, of this city, has given very close attention to the therapeutics of this question, as shown by two papers he read some months ago: One before the New York State Medical Society, January, 1896; and one before the New York Academy of Medicine, February 18, 1896, both papers being published in the *Medical News.*

Other favorable clinical reports which have attracted my attention which may be mentioned here are—in the *Journal of the American Medical Association* for August 15, 1896, by Dr. T. H. Allen; in the *Medical Age* for July 25, 1896, by Dr. F. Spencer Halsey; and in the *Therapeutic Gazette* for September 15, 1896, by Dr. Wm. A. Walker.

In the practice of some friends, who have reported the matter to me for elucidation, this form of diastase seems to have other properties than its action on starch. In one notable case in which pepsin and other methods of treatment failed to give any benefit, although every symptom and test seemed to indicate that it was an undoubted case of albuminous indigestion, the result was not only palliative but curative. I have endeavored to have this matter settled by laboratory experiments, but so far the results have not been entirely conclusive, so any theory on this question for the present must be based mainly upon clinical evidence. The disastase does, as was shown by the experiments referred to, *disintegrate* albumens, but the proteolytic action *apparently* stops short of the production of albumoses and peptones. As to what takes place in the intestinal tract, of course we cannot yet say. It is altogether probable that the benefit in these cases is due to the promptness with which the first period of digestion is carried on and the conversion, instead of fermentation, of starchy foods, leaving the second process, the acid peptic digestion, to go on normally without being interfered with by deleterious products, and the partial disintegration above referred to doubtless promotes the activity of the gastric juice by giving it freer access to the particles of albumen.

We should always bear in mind that we have from thirty to forty minutes after the close of an ordinary meal in which the action of ptyalin or diastase will continue before the acidity of the stomach contents reaches the point at which such converting power is impaired or destroyed. The proper theory for the administration of diastase is that it supplements the ptyalin of the saliva, and the more thorough the preliminary digestion in the stomach, the less work there is to be done in the duodenum. The formerly prevalent theory, that pancreatic extracts and diastase ought in some mysterious way to find their way through the stomach into the duodenum, and there begin their work, is too absurd to be entertained. (See Dr. Walker's article above referred to.) I am indebted to Dr. Henry Dwight Chapin for information in this connection (*N. Y. Medical Journal*, September 16, 1893), the results of some elaborate experiments which he had made in the Post-Graduate Laboratory in 1893. The experiments were made with a product containing diastase; the stomach was washed out, and after the subject had been properly fed, the diastase was administered in certain cases and omitted in others. The report made to Dr. Chapin by the chemist in charge of the work was, that when the stomach was emptied, forty minutes after the administration, the percentage of food remaining in the stomach at this time averaged, when diastase was not given, 52.02 per cent.; with diastase, 20.2 per cent., showed very conclusively the action of the diastase on the stomach. An analysis of the solid food remaining in the stomach showed that when diastase had not been administered, 7.02 per cent. were undissolved; when it was given, only 3.45 per cent.

Experiments with diastase have not up to this time been as satisfactorily conducted as those made with pepsin, for the reason that dastase is a sensitive body, and the value of any laboratory or test-tube experiments are apt to be contradictory, unless proper precautions are preserved as to the degree of heat used, and the various brands of starch found in the market vary in reaction and in their sensible properties. It is to be hoped that we will soon have definite tables in use for this work, so that all experiments being made by a uniform standard, the results can be more intelligently compared and studied.

I might add, in conclusion, that the therapeutic properties of diastase have not yet been as thoroughly investigated as could be desired. A good deal of careful work is now being done by investigators in this and other countries, and I am confident that the literature of the subject will be enriched very greatly in the near future.

So far little has been done with diastase in practical medicine beyond its use in typical cases of amylaceous indigestion. What we may expect from its employment in partially converting the starch of barley water for infant feeding remains yet to be determined, but I am very hopeful of its use in such cases. The fact of the casein of cow's milk being so much more dense and liable to form tough curds than human milk, has led me to hope that if we partially convert the starch of the barley water it will become not only a mechanical diluent for milk, but a readily absorbable food as well, which will nourish and not give rise to fermentation and flatulence.

Some time since I witnessed several experiments in bread-making made by Mr. C. Von Egloffstein, then of Yonkers, now of Brooklyn, N. Y., to determine the value of diastase in rendering bread more soluble. His conclusions were that the soluble matter in ordinary bread in water at a temperature of 100° F. represented at the end of an hour 15 per cent., whereas when a proper amount of a product containing diastase was added to the bread before baking, the soluble matter under the same conditions was 40 per cent.

It is more than probable that diastase will play an important part in the therapeutics of the future.

*102 W. 93d Street.*

